# Editorial: Neurocognitive disorders in the community: a global perspective

**DOI:** 10.3389/frdem.2025.1663449

**Published:** 2025-09-09

**Authors:** Panagiotis Alexopoulos, Katherine Lawler, Chukwuanugo Ogbuagu, Arianna Almirall Sanchez, Asri Maharani

**Affiliations:** ^1^Mental Health Services, Faculty of Medicine, School of Health Sciences, University of Patras, Patras, Greece; ^2^Global Brain Health Institute, School of Medicine, Trinity College Dublin, The University of Dublin, Dublin, Ireland; ^3^Department of Psychiatry and Psychotherapy, Klinikum Rechts der Isar, Technical University of Munich, Munich, Germany; ^4^Patras Dementia Day Care Centre, Patras, Greece; ^5^Wicking Dementia Research and Education Centre, University of Tasmania, Hobart, TAS, Australia; ^6^School of Allied Health, Human Services and Sport, La Trobe University, Melbourne, VIC, Australia; ^7^Nnamdi Azikiwe University Teaching Hospital, Nnewi, Nigeria; ^8^Global Brain Health Institute, University of California, San Francisco, San Francisco, CA, United States; ^9^Department of Public Health and Primary Care, School of Medicine, Trinity College Dublin, Dublin, Ireland; ^10^Mental Health Research Group, Division of Nursing, Midwifery and Social Work, School of Health Sciences, University of Manchester and Manchester Academic Health Science Centre (MAHSC), Manchester, United Kingdom

**Keywords:** dementia, brain health, collaborative services, equity, prevention

The prevalence of neurocognitive disorders is projected to rise substantially, particularly in low- and middle-income countries (LMICs) (Sachdev et al., 2014; Nichols et al., 2022). Interestingly, the development, clinical expression and progression of brain pathologies causing neurodegenerative conditions, are influenced by modifiable risk factors like low cognitive brain reserve, sensory loss and hypertension, which account for approximately 45% of dementia cases (Livingston et al., 2024). The deciphering of this complex interplay triggers a shift from disease-based management strategies to brain health-promoting ones (Fox et al.). This Research Topic of papers explores global approaches to brain health promotion and dementia prevention, the impact of neurocognitive disorders on communities at individual and societal levels, and innovative approaches to diagnostic and post diagnostic care services addressing inequities.

The rapidly increasing number of people with cognitive decline renders radical changes in the paradigm of care services for neurocognitive disorders inevitable. Three papers in this Research Topic highlight that in low-resource settings memory clinics and old-age mental health services are scarce and primary healthcare providers do not routinely carry out cognitive screening in older people (Ogbuagu et al., 2023; Fox et al.; Ali et al.; Novotni et al.). Collaborative healthcare services, bridging primary healthcare services with secondary or tertiary ones, may pragmatically address the changing and complex healthcare needs of people with neurocognitive disorders (Novotni et al.; Aggeletaki et al.; Tunnard et al.), and the needs of their care partners (Kennedy et al.). They are based on multiprofessional teams who are aware of the particularities of the local setting. In this Research Topic, three living labs (Wolff et al., 2024) of such collaborative community services are presented, mainly a multiprofessional case management and e-psychoeducation model of services delivered by mobile teams (Novotni et al.) and two telehealth-based memory clinic models embedded within primary healthcare services in low-resource settings (Aggeletaki et al.).

Considering the significant impact of modifiable risk factors on the development of cognitive decline in aging and the still small effect sizes of potentially disease modifying drugs, dementia prevention remains a priority and was the primary focus of two papers in the Research Topic (Andreoletti and Blasimme; Fox et al.). Indeed, dementia prevention has been included as a strategic goal in the National Dementia plans of several European and North American countries, while in LMICs like Pakistan, no such plans have been enacted at all (Andreoletti and Blasimme; Ali et al.). In both papers the benefits of evidence-based population-wide approaches to dementia prevention over those relying on lifestyle interventions alone are underscored. Nonetheless, even if included in National Dementia plans, prevention actions are commonly depicted in very general terms and their implementation is undermined by staff shortages, lack of interest of healthcare professionals, pervasive stigma pertaining to aging and dementia and widespread misconceptions regarding the nature of age-related cognitive decline (Fox et al.).

Modifiable cognitive decline risk factors are related to cardiovascular, sensory, mental and physical health, as well as to societal determinants of brain health which makes multiprofessional cooperation essential for implementing dementia prevention strategies (Gorelick and Sorond, 2024). Interestingly, the ties of collaborative community healthcare services to local contexts may facilitate localized approaches to addressing dementia risk factors, brain health promotion and destigmatization of aging and dementia (Fox et al.). Indeed, the impact of each dementia risk factor may vary across different contexts, considering for instance the high rates of brain injuries in Pakistan because of lack of road regulations, domestic and sexual violence or the better memory function of older rural-dwellers compared to urban-dwellers in Southern Nevada in the United States, even though rural living commonly poses putative risk for cognitive decline and dementia (Ali et al.; Miller et al.). This variability warrants in-depth investigation and should inform local awareness raising campaigns, prevention and care. In addition, the central role of primary healthcare professionals in collaborative healthcare services enables a “one-stop-shop” approach to the management of several risk factors of cognitive decline (Aggeletaki et al.).

Collaborative community healthcare services are increasingly based on telemedicine, with technology a key focus of two papers in the Research Topic (Aggeletaki et al.; Tunnard et al.). E-health tools facilitate communication between collaborating healthcare professionals serving at spatially distant services, between service users and healthcare professionals, distant monitoring of symptoms and behavior of older people living alone, support for their informal care partners and/or assessment and decision-making for people with dementia living in care homes (Schaller et al., 2015; Vuong et al., 2015) (Tunnard et al.). For instance, e-health tools can be used by the staff of care homes to communicate the symptoms of the residents through video consultations, safeguarding appropriate prescribing, advance care planning and reduction of hospitalizations (Tunnard et al.).

Although telemedicine contributes to the quality of dementia care, it can also lead to inequities in access and usage (Wolff et al., 2024). Equity promoting e-health solutions was a recurring theme in this Research Topic of papers. The development of practical guidelines for equitable e-health solutions for dementia care in the UK is depicted (Wolff et al., 2024), so does a tablet-based, brain health assessment tool which was adapted to the cultural nuances of communities in Southeast Nigeria (Ogbuagu et al.).

Despite efforts reported in this Research Topic, there remains a strong and ongoing need for action to tackle inequities at local and national levels. Work relating to National Dementia Action Plans remains of foundational importance. Yet every dementia researcher and clinician could also consider inequity when planning future work and/or in their everyday clinical practice. What partnerships could be developed, big or small, to nudge toward health equity for people living with dementia or cognitive impairment? What tweaks could be made to a research project to allow access for people living in low-resource settings? In addition, it is urgent that researchers and clinicians from LMICs are resourced or supported to shape innovative dementia research and care agendas in their countries rather than having to adapt work from other parts of the world later.

In conclusion, the papers included in this Research Topic provide valuable insights into the challenges of care and prevention of age-related cognitive decline in diverse communities worldwide. They can inform the development of pragmatic community services that are adjusted to local contexts and meet local brain health needs, and fuel future research endeavors.
